# Comparison of Ideal vs. Actual Body Weight Dosing of Intravenous Immunoglobulins for Immune Thrombocytopenia: A Retrospective Analysis

**DOI:** 10.1155/ah/8770122

**Published:** 2025-12-01

**Authors:** Hamdi Lababidi, Valassia Antigone Theocharides, Jimmy Gonzalez

**Affiliations:** ^1^ Central Michigan University College of Medicine, Mount Pleasant, Michigan, USA, cmich.edu; ^2^ Ernest Mario School of Pharmacy, Rutgers, The State University of New Jersey, Piscataway, New Jersey, USA, rutgers.edu; ^3^ Hackensack Meridian Health Jersey Shore University Medical Center, Neptune, New Jersey, USA

**Keywords:** dosing strategy, ideal body weight, immune thrombocytopenia, IVIG

## Abstract

**Introduction:**

Intravenous immunoglobulin (IVIG) is a weight‐based therapy used to treat immune thrombocytopenia (ITP). Although pharmacokinetic data support the use of ideal body weight (IBW)–based dosing, clinical outcomes for this dosing strategy are lacking.

**Materials and Methods:**

This retrospective, multicenter chart review was conducted across five institutions compared clinical outcomes and costs for patients with ITP treated with IVIG dosed via actual body weight (ABW) or IBW. Data were collected from November 1, 2019 to April 10, 2024. The primary outcome was early platelet response, and secondary outcomes included length of stay (LOS) and increase in platelet count.

**Results:**

A total of 94 patients were included for analysis. No significant differences were noted between the ABW and IBW groups for early platelet response (59.1% vs. 47.2%, *p* = 0.466), LOS (9 vs. 6 days, *p* = 0.111), and the increase in platelet count (54.5 vs. 18.5 × 10^9^/L, *p* = 0.681). The estimated average cost of IVIG per patient was $5231.87 lower for the IBW group.

**Conclusions:**

The utilization of IBW‐based dosing of IVIG for ITP treatment was not associated with a change in early platelet response, LOS, or increase in platelet count compared with ABW‐based dosing but was associated with cost savings.

**Summary**

• Treatment outcomes using IBW vs. ABW dosing strategies of IVIG were compared for managing ITP.
• Early platelet response and length of stay were similar between IBW and ABW dosing, though the estimated average IVIG cost per patient was $5231.87 lower in the IBW group.
• IBW dosing of IVIG for ITP may provide similar clinical outcomes with lower treatment costs relative to ABW dosing.

## 1. Introduction

Intravenous immunoglobulin (IVIG) is a pooled, plasma‐derived antibody product dosed as a weight‐based therapy to treat a variety of disorders, including immune deficiencies, autoimmune disorders, and inflammatory disorders [1, 2]. IVIG is a commonly recommended agent alongside corticosteroids for the treatment of ITP, an autoimmune disorder involving the destruction of platelets and impaired platelet production, particularly when a rapid rise in platelet count is clinically necessary [3]. The mechanisms of IVIG in the treatment of ITP may include competitive inhibition of the neonatal Fc receptor and acceleration of antiplatelet antibody elimination thus reducing thrombocytopenia and bleeding risk [2, 4].

Past clinical research assessed the efficacy of IVIG via actual body weight (ABW) dosing [5]. However, ABW‐based dosing results in obese patients (body mass index [BMI] > 30 kg/m^2^) receiving numerically higher IVIG doses and potentially incurring more adverse events (AEs). IVIG has a low volume of distribution of 0.02–0.05 L/kg and distributes throughout the intravascular and extravascular spaces with minimal penetration into lipophilic tissue [1, 6]. Thus, ideal body weight (IBW)–based dosing may be more appropriate given additional dosing administered to obese patients may increase the risk of AEs with uncertain effect on clinical outcomes. Furthermore, IBW‐based dosing may provide institutional cost savings due to the reduction in IVIG utilization.

Previous retrospective studies [6–8] assessing various dosing strategies of IVIG compared clinical outcomes such as hospital readmission rates, length of stay (LOS), hospital mortality, infection rates, and various AEs. Not only did these studies find no statistically significant differences in favor of ABW‐based dosing, but significant reductions in LOS and acute kidney injury rates were associated with alternative dosing strategies (IBW and/or adjusted body weight). Furthermore, institutional cost savings ranging from $14,000 to $375,198.10 were associated with these alternative dosing strategies. Other publications of IVIG in patients with ITP include pediatric studies: a meta‐analysis [9] comparing low‐ and high‐dose IVIG and an observational study [10] evaluating high‐dose IVIG. Of note, the meta‐analysis demonstrated no significant differences in effective rate, durable remission, or platelet count improvement between dosing strategies, while demonstrating a lower frequency of AEs in the low‐dose group [9]. However, there is a lack of evidence establishing an optimal IVIG dosing strategy for adult patients with ITP.

Based on such literature, a large academic hospital network in the Northeastern United States revised medication guidelines and the electronic health records medication orderable to adopt IBW‐based dosing as the default dosing strategy for IVIG. However, further research is needed to compare the two dosing strategies. There is no published study comparing weight‐based dosing strategies in patients with ITP specifically, and data comparing ITP‐specific clinical outcomes are lacking. The purpose of this study is to compare the clinical outcomes between ABW‐ and IBW‐based dosing of IVIG in the treatment of ITP as well as to compare the costs associated with the two dosing strategies.

## 2. Methods

### 2.1. Study Design

This was a multicenter, retrospective analysis of hospitalized adult patients across five hospitals in a multiple hospital network after the defaulting of IBW‐based IVIG dosing through the electronic health record system; prescribers were still able to modify dosing to other strategies as clinically warranted. Patients were included if they received IVIG treatment (either ABW‐ or IBW‐based strategies) for ITP between November 1, 2019, and April 10, 2024. Two cohorts were identified based on dosing strategy: the ABW and IBW groups. IBW was calculated using the following equation for males: 50 + [2.3 × (# of inches over 5 feet)]. For females, the equation was 45.5 + [2.3 × (# of inches over 5 feet)]. Patients who were less than 22 years old, pregnant, received IVIG via alternative dosing strategies (e.g., adjusted body weight, order‐specific), received IVIG for a non‐ITP indication, or for whom the assessment of early platelet response was impaired were excluded. Criteria for impaired assessment of early platelet response included the receipt of a different IVIG dose too soon (prior to post‐IVIG platelet labs or < 7 days after baseline platelet labs) or lack of post‐administration platelet labs. Only the first documented episode of ITP treated with IVIG was considered for inclusion. Orders continuing the same doses as previous orders without changes in frequency were grouped into single IVIG encounters. The study was approved by the institutional review boards as expedited research.

### 2.2. Outcomes

The primary outcome was early platelet response, defined as a post‐administration platelet count measured 7 days after IVIG initiation of ≥ 30 × 10^9^/L and at least doubling from the baseline platelet count. If post‐administration platelet counts were unavailable at 7 days, then the nearest platelet counts < 7 days after baseline counts were used. Secondary outcomes assessed included the postadministration platelet count and increases in platelet count from baseline to post‐administration review, time between platelet assessments, and LOS.

Covariates of interest to clinical response were assessed and included the severity of bleeding during the hospital stay, race, percentage of post‐administration platelet labs obtained 7 days after baseline, percentage of baseline platelets < 30 × 10^9^/L, platelet transfusion receipt, packed red blood cell transfusion receipt, BMI, COVID‐19 status, and concomitant corticosteroid use. AEs assessed were gastrointestinal events (nausea, vomiting, and/or diarrhea), acute kidney injury, infection, and venous thromboembolism.

Additionally, the number of doses and total grams ordered per encounter were assessed. Individual encounters represented individual patients’ combined number of grams of IVIG ordered for patients receiving multiple IVIG infusions. To estimate realized cost savings per patient, the total grams of IVIG for each group and the corresponding costs were calculated using an average wholesale price of $1643.04 per 10‐g vial of Gamunex‐C (Grifols Therapeutics Inc, Research Triangle Park, NC, USA) and $1937.40 per 10‐g vial of Gammagard (Takeda Pharmaceuticals U.S.A. Inc, Lexington, MA, USA) [11, 12]. Potential savings were also calculated by calculating the hypothetical number of IVIG grams ordered if the patients in the ABW group were dosed via IBW (rounding each total dose to the nearest 5 g to better represent clinical practice), calculating the alternative cost, and comparing to the realized cost of IVIG incurred by the ABW group. A subgroup cost analysis of patients with BMI > 30 kg/m^2^ was conducted as well.

### 2.3. Statistical Analysis

Descriptive statistics (mean, standard deviation, median, interquartile range, percentage, frequency) were reported for demographic and clinical outcome data. Wilcoxon rank‐sum tests were used to compare continuous data, and Fisher’s exact tests were used to compare categorical data. *p* values of < 0.05 were considered statistically significant. A subgroup analysis was performed for patients who did not receive packed red blood cell transfusions during their hospital stay.

A logistic regression analysis was also performed to assess the effects of select covariates on platelet response (dosing strategy, platelet transfusion receipt, baseline platelet count, and corticosteroid receipt). SPSS Statistics 28 (IBM, Aromonk NY) and SAS software (SAS Institute Inc, Cary, NC) were used to perform the statistical analyses.

## 3. Results

### 3.1. Patients

**Figure 1:**
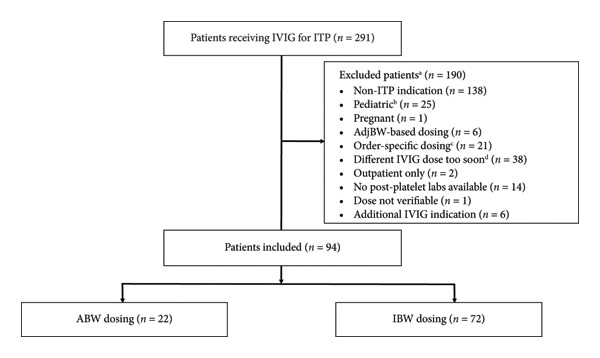
Enrollment flowchart. ^a^Some patients met multiple exclusion criteria. ^b^Age ≤ 21 years. ^c^Dosing strategy differs from ABW/IBW/adjusted body weight or if order states “order‐specific.” ^d^Prior to post‐platelet labs or < 7 days after baseline platelet labs. Abbreviations: IVIG, intravenous immunoglobulin; ITP, immune thrombocytopenia; AdjBW, adjusted body weight; AIHA, autoimmune hemolytic anemia; ABW, actual body weight; IBW, ideal body weight.

A total of 291 patients receiving IVIG for ITP were identified and assessed for inclusion of which 94 patients were included for analysis: 22 in the ABW group and 72 in the IBW group (Figure 1). No patients had concomitant hemophilia A, hemophilia B, or von Willebrand disease. No significant differences were noted at baseline for age, ABW, IBW, BMI, sex, ethnicity, and prescriber type (hematology/oncology specialist vs. other). However, the ABW group possessed a significantly higher percentage of Black and African American patients (27.3% vs. 6.9%, *p* = 0.018). Table 1 outlines the baseline characteristics.

**Table 1:** Baseline characteristics.

Variable, mean (SD)	ABW group (*n* = 22)	IBW group (*n* = 72)	*p* value
Age (years)	64.3 (17.4)	63.8 (17.1)	0.908
Height (cm)	169.4 (13.4)	169.0 (11.2)	0.900
ABW (kg)	83.2 (25.4)	87.5 (22.9)	0.460
IBW (kg)	63.1 (14.0)	63.3 (11.4)	0.792
BMI (kg/m^2^)	28.6 (6.3)	30.5 (7.0)	0.467
Sex (*n* (%))			
Female	11 (50)	32 (44.4)	0.807
Male	11 (50)	40 (55.6)	—
Race, *n* (%)			
AIAN	0 (0)	1 (1.4)	1.000
Asian	0 (0)	2 (2.8)	1.000
NHOPI	0 (0)	0 (0)	N/A
Black/African American	6 (27.3)	5 (6.9)	0.018
White	15 (68.2)	59 (81.9)	0.232
Other	1 (4.6)	5 (6.9)	1.000
Ethnicity, *n* (%)			
Hispanic/Latino	2 (9.1)	6 (8.3)	1.000
Not Hispanic/Latino	19 (86.4)	65 (90.3)	—
Unknown/not reported	1 (4.6)	1 (1.4)	0.415
Hematology/oncology prescriber, *n* (%)	19 (86.4)	47 (65.3)	0.067
Platelet count (× 10^9^/L), median (IQR)	17.5 (8, 28)	10.5 (5, 21)	0.070
Serum creatinine (mg/dL), median (IQR)	0.86 (0.66, 1.27)	0.95 (0.74, 1.22)	0.386
Blood products, *n* (%)			
Platelet transfusion	15 (68.2)	55 (76.4)	0.577
Packed red blood cell transfusion	10 (45.5)	25 (34.7)	0.451
COVID‐19^†^, *n* (%)	1 (6.3)	7 (11.9)	1.000

*Note:* IQR, interquartile range.

Abbreviations: ABW, actual body weight; AIAN, American Indian or Alaska Native; BMI, body mass index; IBW, ideal body weight; NHOPI, Native Hawaiian or Other Pacific Islander; SD, standard deviation.

^†^Only applicable to patients hospitalized after March 2020.

### 3.2. Clinical Outcomes

For this study, platelet counts listed as ≤ 3 × 10^9^/L in the electronic medical record were counted as 3 × 10^9^/L, and those listed as < 2 × 10^9^/L were counted as 2 × 10^9^/L. Serum creatinine levels listed as < 0.3 mg/dL were counted as 0.3 mg/dL. The primary outcome of early platelet response was not significantly different between the ABW and IBW groups (59.1% vs. 47.2%, *p* = 0.466). Secondary outcomes between the two groups were also not significantly different (Table 2); however, the ABW group had a numerically higher post‐administration platelet count (74 vs. 45.5 × 10^9^/L, *p* = 0.084). Furthermore, there were no significant differences in AEs between the two groups (Table 2) or platelet response rates among specified subgroups (Table 3). In terms of median change in serum creatinine, there was no significant difference between the two groups (−0.27 vs. −0.03 mg/dL, *p* = 0.477) in 93 patients (one patient lacked a post‐administration serum creatinine). In the subgroup analysis of patients who did not receive packed red blood cell transfusions during their hospitalization (Table 2), the ABW group possessed a significantly higher median LOS (9 vs. 4 days, *p* = 0.024).

**Table 2:** Primary and secondary outcomes.

Variable	ABW group (*n* = 22)	IBW group (*n* = 72)	*p* value
Primary outcome, *n* (%)			
Early platelet response^†^	13 (59.1)	34 (47.22)	0.466
Secondary outcomes, median (IQR)			
Posttreatment platelet count (× 10^9^/L)	74 (27, 155)	45.5 (20, 117.5)	0.084
Increase in platelet count (× 10^9^/L)	54.5 (−2, 136)	18.5 (6.5, 94.5)	0.681
Time between platelet assessments (days)	4.5 (3, 7)	5 (3, 7)	0.853
Length of stay, days	9 (4, 19)	6 (3, 13)	0.111
IVIG characteristics			
Brand of IVIG product, *n* (%)			
Gamunex‐C	22 (100)	65 (90.3)	0.194
Gammagard SD	0 (0)	7 (9.7)	—
Dose analysis, median (IQR)			
Number of divided doses	2 (2, 4)	2 (1, 2)	0.046
Total grams ordered	145 (50, 200)	100 (62.5, 130)	0.075
Adverse events, *n* (%)			
Gastrointestinal events^‡^	6 (27.3)	6 (8.3)	0.030
Acute kidney injury^§^	1 (4.6)	12 (16.7)	0.287
Infection^§^	3 (13.6)	18 (25.0)	0.527
Venous thromboembolism^§^	0 (0.0)	1 (1.4)	1.000
Disseminated intravascular coagulation^§,¶^	0 (0.0)	1 (1.4)	1.000
Fever^§,¶^	0 (0.0)	1 (1.4)	1.000
Mild headache^§,¶^	1 (4.6)	0 (0.0)	0.234
Hypertension^§,¶^	0 (0.0)	1 (1.4)	1.000

**No packed red blood cell transfusion subgroup analysis**	**ABW group (*n* = 12)**	**IBW group (*n* = 47)**	**p** **value**

Primary outcome, *n* (%)			
Early platelet response^†^	8 (66.7)	26 (55.3)	0.532
Secondary outcomes, median (IQR)			
Post‐treatment platelet count (× 10^9^/L)	92 (48, 141.5)	64 (26, 139)	0.283
Increase in platelet count (× 10^9^/L)	61.5 (−1.5, 129)	45 (9, 127)	0.858
Time between platelet assessments (days)	4.5 (3.5, 7)	5 (2, 6)	0.339
Length of stay (days)	9 (5, 13.5)	4 (2, 8)	0.024

*Note:* IQR, interquartile range; IVIG, intravenous immunoglobulin.

Abbreviations: ABW, actual body weight; IBW, ideal body weight.

^†^Platelet count ≥ 30 × 10^9^/L AND at least doubling from baseline at 1 week, or latest available count prior to 1 week.

^‡^Nausea/vomiting/diarrhea within 24 h of beginning of IVIG administration, regardless of causality attribution.

^§^New onset or worsened by IVIG.

^¶^Considered if providers blame adverse event on IVIG.

**Table 3:** Platelet response rate among specific subgroups.

Variable, *n* (%)^‡^	ABW group	IBW group	*p* value
Bleeding^†^			
Major bleeding	4 (50.0)	12 (54.6)	1.000
Minor bleeding	7 (70.0)	12 (40.0)	0.148
No bleeding	2 (50.0)	10 (50.0)	1.000
Black/African American race	4 (66.7)	2 (40.0)	0.567
White race	8 (53.3)	24 (45.8)	0.773
Platelet labs at 7 days post‐IVIG	8 (36.4)	24 (46.2)	0.437
Baseline platelets < 30 × 10^9^/L	12 (70.6)	30 (50.9)	0.176
Platelet transfusion	8 (53.3)	23 (41.8)	0.560
Packed red blood cell transfusion	5 (50.0)	8 (32.0)	0.444
BMI ≥ 30 kg/m^2^	7 (70.0)	14 (48.3)	0.290
COVID‐19 status			
Positive	1 (100)	2 (28.6)	0.375
Negative	9 (60.0)	25 (48.1)	0.560

*Note:* IVIG, intravenous immunoglobulin.

Abbreviations: ABW, actual body weight; BMI, body mass index; IBW, ideal body weight.

^†^Major = (1) WHO grade 3 or 4 bleeding, (2) Buchanan severe grade, (3) Bolton, Maggs, and Moon “major bleeding,” (4) IBLS grade 2 or higher, or (5) life‐threatening bleeding or intracerebral hemorrhage; Minor = bleeding not meeting criteria for major bleeding; assessed at any time during index hospitalization.

^‡^Values are reported by individual subgroups, each with a different total sample size.

An analysis of concomitant corticosteroid use determined no significant differences in corticosteroid receipt (90.9% vs. 93.0%, *p* = 0.667) (Supporting Table 1). A logistic regression analysis of platelet response found no significant impact of dosing strategy (adjusted odds ratio, aOR: 0.404; 95% CI: 0.131, 1.246) or corticosteroid receipt (aOR: 1.578; 95% CI: 0.274, 9.100); however, platelet transfusion receipt (aOR: 0.244; 95% CI: 0.077, 0.774) and baseline platelet count (aOR: 0.942; 95% CI: 0.907, 0.977) were associated with decreased odds of platelet response after adjustment for other covariates (Table 4).

**Table 4:** Association between dosing strategy and select covariates with platelet response.

	Unadjusted OR	(95% CI)	aOR	(95% CI)
ABW strategy	Ref	—	Ref	—
IBW strategy	0.619	0.235, 1.630	0.404	0.131, 1.246
Platelet transfusion	0.397	0.151, 1.049	0.244	0.077, 0.774
Baseline platelet count (× 10^9^/L)	0.956	0.926, 0.987	0.942	0.907, 0.977
Corticosteroid receipt	1.397	0.295, 6.617	1.578	0.274, 9.100

Abbreviations: ABW, actual body weight; aOR, adjusted odds ratio; CI, confidence interval; IBW, ideal body weight; OR, odds ratio; Ref, reference.

### 3.3. Cost Analysis

Based on the average wholesale prices of the two IVIG formulations used (as of May 10, 2023), the use of IBW‐based dosing netted an average savings of $5231.87 per patient (Table 5). A subgroup analysis of obese patients (BMI > 30 kg/m^2^) demonstrated a greater average savings of $11,259.67 per patient using IBW‐based dosing (Supporting Table 2). Furthermore, every patient in the ABW group had been dosed via IBW, and then, the additional savings incurred were estimated to be $124,871.04 in total.

**Table 5:** Cost analysis.

Variable	ABW group (*n* = 22)	IBW group (*n* = 72)
Total Gamunex‐C (grams)	2925	6661
Total Gamunex‐C ($)	480,589.20	1,094,428.94
Total Gammagard (grams)	0	525
Total Gammagard ($)	0	101,713.50
Total IVIG (grams)	2925	7186
Total cost ($)	480,589.20	1,196,142.44
Average cost per patient ($)	21,844.96	16,613.09

*Note:* Total grams calculated based on the total grams ordered per encounter, with single encounters representing single patients receiving single or multiple IVIG doses. Cost was calculated based on average wholesale prices of $1643.04 per 10‐g vial of Gamunex‐C and $1937.40 per 10‐g vial of Gammagard as of May 10, 2023.

Abbreviations: ABW, actual body weight; IBW, ideal body weight; IVIG, intravenous immunoglobulin.

## 4. Discussion

Optimal dosing of IVIG and subcutaneous immunoglobulin remains uncertain. The initial report of IVIG’s immunomodulatory effects [10] was followed by controlled clinical analyses in ITP and other similar autoimmune conditions [13]. These reports, however, were not followed by dose‐finding studies. To our knowledge, this is the first study comparing ABW‐ and IBW‐based dosing of IVIG specifically for the treatment of ITP, and our findings add to current efforts to clarify IVIG dosing strategies [9]. This study found that IBW‐based dosing of IVIG was not associated with significant differences in early platelet response rates, changes in platelet count, LOS, or AEs across multiple sites. Of note, early platelet response was chosen as the primary outcome because the time to initial response of IVIG is 1–3 days and peak response occurs in 2–7 days [14]. The assessment of post‐IVIG administration platelets at 7 days was consistent with these pharmacokinetic parameters and with clinical guidelines [3]. Although post‐administration platelet counts were significantly higher in the ABW group, this can be explained by the significantly higher baseline platelet counts in the ABW group as the changes in platelet count were not significantly different between the two groups.

Interestingly, the higher baseline platelet counts in the ABW group were associated with nominally decreased odds of response via logistic regression analysis. This effect may be due to the bipartite definition of platelet response whereby higher baseline values were more difficult to demonstrate doubling at the second measurement. Similarly, this measure does not capture significant quality of life improvements associated with platelet counts > 50 × 10^9^/L, though we found no statistical difference between the two groups. Additionally, platelet transfusion, after adjustment for other covariates, was associated with >80% decreased odds of early platelet response, which may reflect the severity of disease presentation necessitating more aggressive management.

Clinical manifestations of bleeding were assessed at any time during index hospitalizations and were not found to be significantly different between the two groups. Regarding racial differences, it was noteworthy that the percentage of Black/African American patients was significantly higher in the ABW group. Clinicians may have opted to dose these patients via ABW given that this patient population has on average a higher lean body mass [15]. To an extent, BMI ranges that are considered obese in the general population may not be as predictive of obesity in this population. Additional subgroup analyses did not yield significant differences (Table 3).

In the subgroup analysis of patients who did not receive packed red blood cell transfusions, there was a significant decrease in LOS in the IBW group possibly indicating an association between this factor and this secondary outcome. In terms of concomitant corticosteroids, there were no significant differences observed in corticosteroid receipt, dosing, and timing relative to IVIG initiation. This conclusion was further reinforced by logistic regression analysis, and so this factor did not appear to result in confounding.

The decision to utilize IBW‐ over ABW‐based dosing led to average estimated savings of $5231.87 per patient overall, and $11,259.67 per patient with BMI > 30 kg/m^2^. Had all patients in the ABW group been dosed via IBW, the additional savings incurred were predicted to have been $124,871.04 in total. It was interesting to note that this figure was as large as $104,333.04 for the subgroup of patients with BMI > 30 kg/m^2^, even though these patients comprised only 39 out of 94 patients in the study. This suggests that IBW‐based dosing may be especially cost‐effective in patients with BMI > 30 kg/m^2^.

A similar study [6] evaluating IVIG across various indications estimated total savings to be $375,198.10 for 297 patients. On a per capita basis, the cost savings associated with IBW‐based IVIG dosing may be more substantial for ITP. Thus, institutions may see potentially large cost savings through the utilization of this dosing strategy which may depend on center volume, indications for use, and patient anthropometrics.

There are several limitations to our study. The study was a retrospective observational chart review which precludes determination of causality between exposure to IBW‐ vs. ABW‐based dosing and clinical outcomes assessed. Likewise, the small sample size introduces the potential for type II error; however, the study included patients from multiple sites within a network to capture as large of a basis during the allotted study time frame. In addition, the electronic health record was unable to inform us of IVIG use outside the network.

In conclusion, the utilization of IBW‐based dosing did not significantly differ from ABW‐based dosing for early platelet response rates and increases in platelet count, LOS, or AEs in adult patients being treated with IVIG for ITP. Substantial cost savings were generated via the utilization of IBW‐based dosing, especially for patients with BMI > 30 kg/m^2^. These findings may assist in implementing strategies to decrease institutional drug expenditure without significantly affecting clinical outcomes. Further research, such as a prospective investigation with a larger sample size or a meta‐analysis, would be needed to further explore differences between these two treatment strategies.

**Nomenclature**
ABW: Actual body weight
AE: Adverse event
BMI: Body mass index
IBW: Ideal body weight
ITP: Immune thrombocytopenia
IVIG: Intravenous immunoglobulin
LOS: Length of stay

## Ethics Statement

All procedures were performed in compliance with relevant laws and institutional guidelines and have been approved by the Hackensack Meridian Health and Rutgers University institutional review boards (IRBs) as expedited research. The Hackensack Meridian Health IRB approved the project on 09/21/2022, and the Rutgers University IRB approved the project on 12/24/2022 (IRB ID: Pro2022‐0661).

## Consent

A waiver of HIPAA authorization and consent was approved due to the retrospective nature of this study.

## Conflicts of Interest

The authors declare no conflicts of interest.

## Author Contributions

Hamdi Lababidi: conceptualization, methodology, software, validation, formal analysis, investigation, data curation, writing–original draft, writing–review and editing, visualization, and project administration. Valassia Antigone Theocharides: investigation, data curation, writing–original draft, writing–review & editing, visualization, and project administration. Jimmy Gonzalez: conceptualization, methodology, software, validation, formal analysis, investigation, data curation, writing–review and editing, visualization, supervision, and project administration.

## Funding

No funding was received for this manuscript.

## Supporting Information

Additional supporting information can be found online in the Supporting Information section.

## Supporting information


**Supporting Information 1** Supporting Table 1. Corticosteroids Received During Encounter. Supporting Table 1 compares the use of corticosteroids among the patients in the ABW and IBW groups throughout the hospital course during which they received IVIG. It analyzes which corticosteroids were administered (dexamethasone, methylprednisolone, prednisone, hydrocortisone) and their median doses. The table further analyzes the total prednisone equivalent given and the timing of dose if the patients received corticosteroids prior to IVIG.

**Supporting Information 2** Supporting Table 2. Cost Subgroup Analysis BMI > 30 kg/m^2^. Supporting Table 2 outlines the cost differences between ABW and IBW IVIG dosing strategies in obese patients with a BMI > 30 kg/m^2^.

## Data Availability

Research data are not shared.
