# Supplementary material for: Comparison of Ideal vs. Actual Body Weight Dosing of Intravenous Immunoglobulins for Immune Thrombocytopenia: A Retrospective Analysis
Source: Adv Hematol. 2025 Dec 1;2025:8770122. doi: 10.1155/ah/8770122 (PMC12669801; doi:10.1155/ah/8770122)
Supplement: Supplementary file 1 — Supporting Information 1 Supporting Table 1. Corticosteroids Received During Encounter. Supporting Table 1 compares the use of corticosteroids among the patients in the ABW and IBW groups throughout the hospital course during which they received IVIG. It analyzes which corticosteroids were administered (dexamethasone, methylprednisolone, prednisone, hydrocortisone) and their median doses. The table further analyzes the total prednisone equivalent given and the timing of dose if the patients received corticosteroids prior to IVIG. [file AH-2025-8770122-s001.docx]

| **Supplemental Table 1.** Corticosteroids Received During Encounter | | |  |
| --- | --- | --- | --- |
| **Variable** | **ABW Group**  **(n = 22)** | **IBW Group**  **(n = 71)^†^** | ***P* Value** |
| **Receipt of any corticosteroid, n (%)** | 20 (90.9) | 66 (93.0) | 0.667 |
| **Type of corticosteroid^‡^** |  |  |  |
| Dexamethasone | 15 (68.2) | 49 (69.0) | 1.000 |
| Methylprednisolone | 6 (27.3) | 24 (33.8) | 0.614 |
| Prednisone | 6 (27.3) | 20 (28.2) | 1.000 |
| Hydrocortisone | 1 (4.6) | 5 (7.0) | 1.000 |
| **Dose of corticosteroid, mg, median (IQR)** |  |  |  |
| Dexamethasone | 160 (60, 160) | 120 (0, 160) | 0.090 |
| Methylprednisolone | 0 (0, 237.5) | 0 (0, 402.5) | 0.680 |
| Prednisone | 0 (0, 90) | 0 (0, 90) | 0.926 |
| Hydrocortisone | 0 (0, 0) | 0 (0, 0) | 0.767 |
| **Total dose in prednisone equivalents, mg** | 1097 (1067, 1446) | 963.8 (533.3, 1166.3) | 0.105 |
| **Receipt prior to initiation of IVIG, n (%)** | 10 (50) | 40 (58.8) | 0.609 |
| ^†^Dosing data missing for one patient  ^‡^Patients may have received more than one type of corticosteroid during an encounter  **Abbreviations**: ABW, actual body weight; IBW, ideal body weight; IQR, interquartile range; IVIG, intravenous immunoglobulin | | |  |
