# Supplementary material for: Comparison of Ideal vs. Actual Body Weight Dosing of Intravenous Immunoglobulins for Immune Thrombocytopenia: A Retrospective Analysis
Source: Adv Hematol. 2025 Dec 1;2025:8770122. doi: 10.1155/ah/8770122 (PMC12669801; doi:10.1155/ah/8770122)
Supplement: Supplementary file 2 — Supporting Information 2 Supporting Table 2. Cost Subgroup Analysis BMI > 30 kg/m2. Supporting Table 2 outlines the cost differences between ABW and IBW IVIG dosing strategies in obese patients with a BMI > 30 kg/m2. [file AH-2025-8770122-s002.docx]

**Supplemental Table 2.** Cost Subgroup Analysis BMI >30 kg/m^2^

| **Variable** | **ABW Group**  **(n = 10)** | **IBW Group**  **(n = 29)** |
| --- | --- | --- |
| Total Gamunex^®^-C, grams | 1,735 | 2,826 |
| Total Gamunex^®^-C, $ | 285,067.44 | 464,323.10 |
| Total Gammagard^®^, grams | 0 | 185 |
| Total Gammagard^®^, $ | 0 | 35,841.90 |
| Total IVIG, grams | 1,735 | 3,011 |
| Total cost, $ | 285,067.44 | 500,165.00 |
| Average cost per patient, $ | 28,506.74 | 17,247.07 |
| Total grams calculated based on the total grams ordered per encounter, with single encounters representing single patients receiving single or multiple IVIG doses. Cost was calculated based on average wholesale prices of $1643.04 per 10-gram vial of Gamunex^®^-C and $1937.40 per 10-gram vial of Gammagard® as of May 10, 2023.  **Abbreviations**: ABW, actual body weight; IBW, ideal body weight; IVIG, intravenous immunoglobulin; BMI, body mass index | | |
